# Morphology-dependent Electrochemical Enhancements of Porous Carbon as Sensitive Determination Platform for Ascorbic Acid, Dopamine and Uric Acid

**DOI:** 10.1038/srep22309

**Published:** 2016-02-29

**Authors:** Qin Cheng, Liudi Ji, Kangbing Wu, Weikang Zhang

**Affiliations:** 1 Key Laboratory for Material Chemistry of Energy Conversion and Storage, Ministry of Education, School of Chemistry and Chemical Engineering, Huazhong University of Science and Technology, Wuhan 430074, China; 2 Department of Gastrointestinal Surgery, Union Hospital, Tongji Medical College, Huazhong University of Science and Technology, Wuhan, 430022, China

## Abstract

Using starch as the carbon precursor and different-sized ZnO naoparticles as the hard template, a series of porous carbon materials for electrochemical sensing were prepared. Experiments of scanning electron microscopy, transmission electron microscopy and Nitrogen adsorption-desorption isotherms reveal that the particle size of ZnO has big impacts on the porous morphology and surface area of the resulting carbon materials. Through ultrasonic dispersion of porous carbon and subsequent solvent evaporation, different sensing interfaces were constructed on the surface of glassy carbon electrode (GCE). The electrochemical behaviors of ascorbic acid (AA), dopamine (DA) and uric acid (UA) were studied. On the surface of porous carbon materials, the accumulation efficiency and electron transfer ability of AA, DA and UA are improved, and consequently their oxidation signals enhance greatly. Moreover, the interface enhancement effects of porous carbon are also controlled by the particle size of hard template. The constructed porous carbon interface displays strong signal amplification ability and holds great promise in constructing a sensitive platform for the simultaneous determination of AA, DA and UA.

Electrochemical sensing has obtained considerable attention due to the following advantages such as high sensitivity, rapidness, good handling convenience, qualification for *in situ* monitoring and excellent compatibility with miniaturization technologies. However, the interface structure and properties are crucial because they have big impacts on the performance of electrochemical sensors. Until now, a large number of researches have proved that interface modification is an efficient strategy to improve the performance of electrochemical sensors^1^,^2^,^3^,^4^. Clearly, preparation of high-performance electrode materials and construction of novel sensing interface are quite important.

In recent years, nanostructured materials with porous structures have been developed rapidly because of their versatile structural diversities and potential physical and chemical functions^5^,^6^,^7^,^8^. Porous carbon, an important branch of porous materials, is highly appealing due to the exceptional characteristics including large surface area, tunable pore size over a wide range, strong accumulation ability and high stability^9^,^10^,^11^. Although numerous methods have been developed for the preparation of porous carbon^12^,^13^,^14^,^15^, template strategy using hard or soft templates has been proven to be the most efficient way to prepare porous carbon with high quality^16^,^17^,^18^,^19^,^20^.

Herein, a series of porous carbon materials were prepared using ZnO nanoparticles with diameters of 15, 30, 50 and 100 nm as the hard templates. Interestingly, we find that the porous structures of the prepared carbon samples are heavily dependent on the particle size of ZnO template, as confirmed from the measurements of scanning electron microscopy, transmission electron microscopy, surface area and pore volume. After that, the obtained porous carbon materials were dispersed into N,N-dimethyl formamide (DMF), and then used to construct various sensing interfaces through solvent evaporation. The electrochemical behaviors of ascorbic acid (AA), dopamine (DA) and uric acid (UA), three kinds of important small biomolecules involved in many physiological processes, were studied. On the surface of porous carbon, the electron transfer of AA, DA and UA is facilitated, and moreover the adsorption ability is also improved obviously. As a result, the constructed sensing interfaces using porous carbon exhibit high electrochemical reactivtiy toward the oxidation of AA, DA and UA, and greatly enhance their oxidation signals. Furthermore, we clearly find that the enhancement effects of the prepared porous carbon are also related to the template size. Based on the structure-controlled interface enhancement ability, a novel electrochemical sensing platform with high sensitivity has been developed for AA, DA and UA.

## Results and Discussion

### Characterization of porous carbon

**Figure 1:**
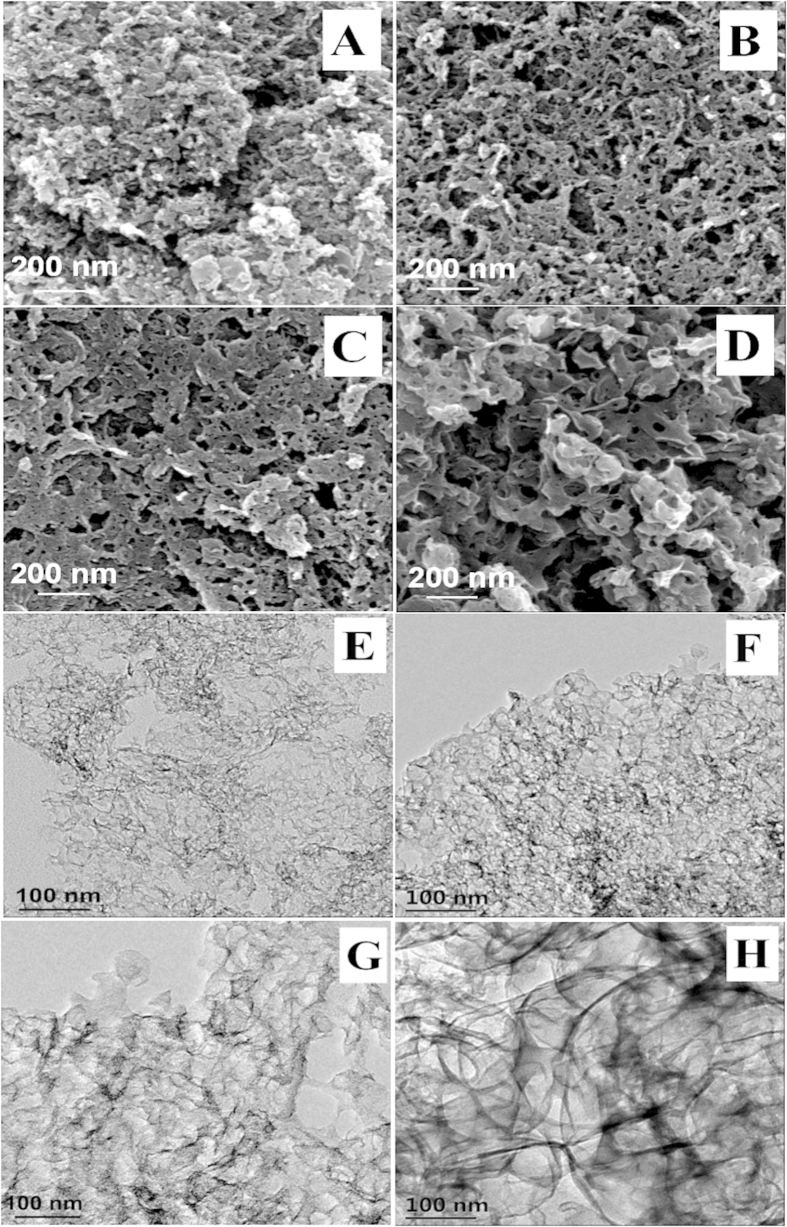
SEM images of PC-15 (**A**), PC-30 (**B**), PC-50 (**C**), PC-100 (**D**) and TEM images of PC-15 (**E**), PC-30 (**F**), PC-50 (**G**), PC-100 (**H**).

The morphology of porous carbon samples was characterized using SEM, and the results were shown in Fig. 1. Porous structures are clearly observed in the samples of PC-15 (A), PC-30 (B), PC-50 (C) and PC-100 (D), and abundant three-dimensional networks are formed. In addition, TEM analysis was employed to provide further information about the porous structures. From Fig. 1, we clearly find that the prepared samples of PC-15 (E), PC-30 (F), PC-50 (G) and PC-100 (H) contain regular and well-developed pores. The pore size is consistent with the particle size of ZnO template, revealing that the site-occupying ZnO nanoparticles work as the templates. In summary, template technology is an efficient strategy to prepare porous carbon materials, and the porous morphology can be easily tuned by varying the particle size of hard template.

The surface area and pore volume of the obtained porous carbon samples were measured through the experiments of adsorption-desorption isotherms, as shown in Figure S1A (Supporting Information). The prepared porous carbon samples exhibit typical IV isotherms with pronounced hysteresis loops. The specific surface area is calculated from the Brunauer-Emmett-Teller (BET) equation, and the pore size distribution (Figure S1B in the Supporting Information) is derived from the desorption branches of the isotherms using the Barrett-Joyner-Halenda (BJH) method. The BET surface areas of PC-15, PC-30, PC-50 and PC-100 are 515.1, 535.0, 516.3 and 501.7 m^2^ g^−1^, with pore volumes of 0.651, 0.966, 0.770 and 0.404 cm^3^ g^−1^. It is apparent that the particle size of hard template has remarkable influences on the properties of porous carbon, and the prepared PC-30 samples possess larger surface area and higher pore volume.

Furthermore, XRD was used to further characterize the structure of porous carbon samples. All the prepared porous carbon materials display similar diffraction features, and two diffraction peaks at about 23.5° and 43.5°, which are the equivalent of hexagonal graphite 002 (2θ = 26°) and 101 (2θ = 43°), are clearly observed (Figure S2A in the Supporting Information). These features indicate that the prepared porous carbon materials are disordered or amorphous. To identify the degree of graphitization, Raman spectra were recorded in Figure S2B (Supporting Information). Two peaks at 1340 cm^−1^ and 1590 cm^−1^ that is assigned to the D band and G band are observed, and the intensity is very close, implying that the obtained porous carbon materials have similar degree of graphitization.

### Electrochemical enhancement of porous carbon

**Figure 2:**
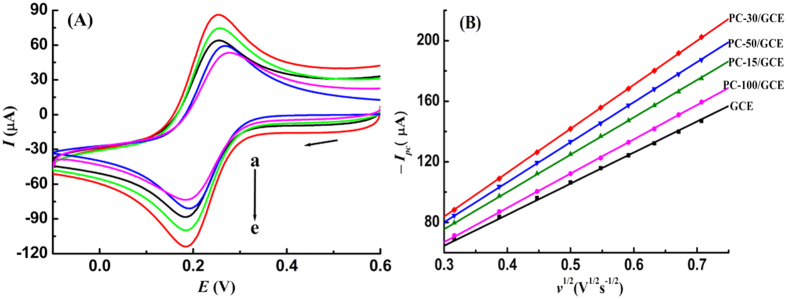
(**A**) Cyclic voltammograms of 5.0 mM K_3_[Fe(CN)_6_] in 1.0 M KCl on GCE (a), PC-100/GCE (b), PC-15/GCE (c), PC-50/GCE (d) and PC-30/GCE (e), scan rate: 100 mV s^−1^; (**B**) Variation of reduction peak currents of K_3_[Fe(CN)_6_] on GCEs with the square root of scan rate.

The electrochemical sensing properties of different porous carbon modified GCEs were examined using the probe of potassium ferricyanide (K_3_[Fe(CN)_6_]). A pair of redox peak with peak potential separation of 88 mV is observed, and the peak currents increase on the surface of porous carbon modified GCEs, compared with those on the bare GCE (Fig. 2A). As gradually increasing the scan rate, the peak currents increase linearly with the square root of scan rate (Fig. 2B), and the peak potentials almost keep stable, suggesting a reversible and diffusion-controlled process. According to the Randles-Sevcik equation, the effective electrode area is individually calculated to be 0.0552, 0.0663, 0.0781, 0.0712 and 0.0609 cm^2^ for GCE, PC-15/GCE, PC-30/GCE, PC-50/GCE and PC-100/GCE. So the modification of porous carbon on GCE surface provides larger electrochemical response area, and besides, the template size has great influences on the electrochemical properties of porous carbon.

**Figure 3:**
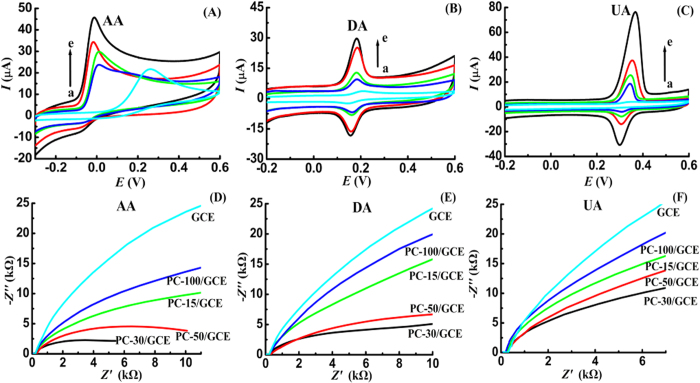
Cyclic voltammograms of 1.0 mM AA (**A**), 50.0 μM DA (**B**) and 50.0 μM UA (**C**) on GCE (a), PC-100/GCE (b), PC-15/GCE (c), PC-50/GCE(d) and PC-30/GCE (e) in pH 6.5 phosphate buffer, scan rate: 100 mV s^−1^; Nyquist impedance plots of 2.5 mM AA (**D**), 200.0 μM DA (**E**) and 200.0 μM UA (**F**) on different GCEs in pH 6.5 phosphate buffer, the fixed potentials for AA, DA and UA were -0.02 V, 0.18 V and 0.34 V, frequency range: 100 kHz to 1 Hz, amplitude: 5 mV.

The elecrochemical behaviors of AA on different GCEs were examined using cyclic voltammetry (CV), and the results were depicted in Fig. 3A. On the surface of bare GCE (curve a), an irreversible oxidation wave is observed for AA in pH 6.5 phosphate buffer, and the peak potential is as high as 0.261V. However, the oxidation peak shifts negatively to −0.017 V, and the peak height enhances obviously on the surface of porous carbon modified GCEs. The lower oxidation peak potential and higher peak currents manifest that the constructed sensing interface using porous carbon has high catalytic ability toward the oxidation of AA. Figure 3B shows the CV responses of DA on the bare GCE (curve a) and porous carbon modified GCEs (curves b to e). In pH 6.5 phosphate buffer, a pair of redox peak with potential of 0.213 V and 0.145 V is observed, and the peak currents increase notably on the surface of porous carbon. The peak current enlargements reveal that the prepared porous carbon materials exhibit strong signal enhancement ability for the oxidation of DA. In addition, the electrochemical responses of UA on different GCEs were also compared using CV in pH 6.5 phosphate buffer. From Fig. 3C, we clearly find that the response signals of UA on the porous carbon modified GCEs enhance obviously, compared with those on the bare GCE surface. In summary, the obtained results from Fig. 3 clearly prove that the prepared porous carbon materials possess remarkable interface enhancement effects, and are more active for the oxidation of AA, DA and UA. Furthermore, the different oxidation signals of AA, DA and UA on the surface of porous carbon indicate that the electrochemical reactivity of porous carbon is controlled by the morphology of pore channels.

To elucidate the origin of enhancement effects for the oxidation signals of AA, DA and UA, the electron transfer behaviors of AA, DA and UA were studied using electrochemical impedance spectroscopy (EIS). As shown in Fig. 3D–F, a semicircle with large diameter is observed on the unmodified GCE surface, and the semicircle decreases greatly on the surface of porous carbon modified GCEs. In Nyquist plot, the diameter of semicircle represents the charge transfer resistance (*R*_ct_) of active species on electrode surface. Consequently, the porous carbon materials facilitate the electron transfer of AA, DA and UA, and greatly enhance their oxidation signals, as confirmed from the obviously-decreased values of *R*_ct_.

In addition, the adsorption behaviors of AA, DA and UA on different GCEs were studied using chronocoulometry to further understand the greatly-increased oxidation signals. In 0.1 M, pH 6.5 phosphate buffer, the curve of charge (*Q*)-time (*t*) is recorded during the potential step, and thereafter transferred to *Q*-t^1/2^ plot, resulting in a straight line. The intercept of *Q*-t^1/2^ plot in the blank phosphate buffer represents *Q*_dl_, the charge devoted to the double-layer capacitance. After individual addition of 1.0 mM AA, 20.0 μM DA or 20.0 μM UA, another *Q*-t^1/2^ straight line is achieved, and the intercept value of *Q*-t^1/2^ plot is the summation of *Q*_dl_ and *Q*_ads_ (the charge derived from the adsorbed species). Thus, the values of *Q*_ads_ on different GCEs are easily calculated, and Table 1 gives the detailed results. Compared with the values on bare GCE, the *Q*_ads_ on porous carbon modified GCEs increases remarkably, demonstrating a more effective accumulation toward AA, DA and UA. Undoubtedly, the resulting oxidation signals of AA, DA and UA are effectively improved by porous carbon. So far, we can conclude that the prepared porous carbon materials display great enhancement effects toward the oxidation of AA, DA and UA due to higher accumulation efficiency and faster electron transfer rate, and moreover, the interface enhancement ability of porous carbon is dependent on the porous morphology.

### Simultaneous determination of AA, DA and UA

**Figure 4:**
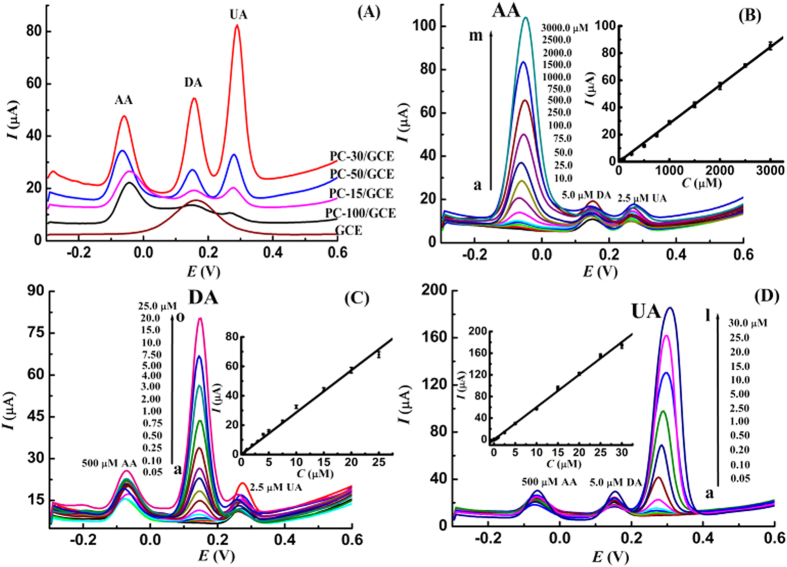
(**A**) DPV curves of 1.0 mM AA, 10.0 μM DA and 10.0 μM UA in pH 6.5 phosphate buffer on different GCEs; (**B**) DPV curves of DA and UA in the presence of different-concentrated AA; (**C**) DPV curves of AA and UA in the presence of different-concentrated DA; (**D**) DPV curves of AA and DA in the presence of different-concentrated UA; Insert plots: corresponding calibration curves; accumulation time: 3 min.

AA, DA and UA are commonly coexisted in biological systems, and their oxidation potentials are close, so it is still full of challenge to develop method for their simultaneous determination. The oxidation responses of AA, DA and UA on different GCEs were studied using differential pulse voltammetry (DPV), and the results were shown in Fig. 4A. On the surface of bare GCE, just a broad oxidation wave is observed for the coexistence of AA, DA and UA in pH 6.5 phosphate buffer. No appearance of independent oxidation peaks indicates that the pristine GCE surface has poor resolution ability toward the oxidation of AA, DA and UA, and thus it is unqualified for the simultaneous determination. However, three well-defined oxidation peaks are observed for AA, DA and UA on the surface of PC-15/GCE, PC-30/GCE, PC-50/GCE and PC-100/GCE. The peak potential difference between AA and DA is as large as 220 mV, and about 135 mV for DA and UA. The large peak potential separation clearly suggests that the mutual interference of AA, DA and UA is very slight. Therefore, the prepared porous carbon materials exhibit excellent resolution ability toward the oxidation of AA, DA and UA, and they are totally qualified for the simultaneous detection of AA, DA and UA. Compared with the peak currents on bare GCE surface, the peak current values of AA, DA and UA enhance obviously on the surface of porous carbon modified GCEs, revealing remarkable signal enhancement effects. In addition, the porous carbon materials that prepared using different-sized ZnO templates display different interface enhancement ability, and the PC-30 is much more active for the oxidation of AA, DA and UA.

From above discussions, it is apparent that the prepared porous carbon materials that using ZnO nanoparticles with size of 30 nm as the hard template exhibit superior electrochemical reactivity toward the oxidation of AA, DA and UA. Consequently, PC-30/GCE is used to construct highly-sensitive sensing platform for the simultaneous determination of AA, DA and UA. Figure 4B illustrates the responses of fixed concentration of DA and UA on PC-30/GCE in the presence of different-concentrated AA. When gradually improving AA concentration from 10.0 to 3000.0 μM, it is found that the oxidation peak currents of AA increase linearly, and the oxidation signals of DA and UA vary slightly. Similar experiments were carried out, and the results were shown in Fig. 4C,D. The obtained results from Fig. 4B–D strongly prove that the oxidation of AA, DA and UA on PC-30/GCE is independent, and their mutual interference is very slight. Moreover, the linear range and detection limit for AA, DA and UA were also examined, and the detailed results were summarized in Table 2. It is apparent that the constructed sensing platform using PC-30 exhibits wide linearity and high sensitivity for the simultaneous determination of AA, DA and UA, as compared with the reported results that listed in Table 3.

The potential interferences on the detection of UA, DA and AA were also studied. No obvious influences on the determination of 500.0 μM AA, 5.0 μM DA and UA are found after addition of 0.01 M glucose, sucrose, sodium benzoate and citric cid; 0.01 M Fe^3+^, Ca^2+^, Mg^2+^, Zn^2+^, Cl^−^, NO_3_^−^ and glycine (signal change lower than 5%).

The reproducibility and stability of PC-30/GCE were also evaluated. For 500.0 μM AA, 5.0 μM DA and UA, the values of relative standard deviation (RSD) are 4.6%, 4.3%, and 4.4% for eleven successive measurements using one PC-30/GCE. After one-week of storage, the signal change is just 4.1%, 3.8%, and 4.7% for AA, DA and UA. The low RSD value and signal change reveal that the developed PC-30/GCE has good reproducibility and stability. In addition, the reproducibility between multiple PC-30/GCEs was also examined, and the values of RSD was lower than 5%.

In order to testify its practical application, this new sensing system was employed in the sample analysis of blood serum. After addition of 1.00 mL sample solution into 9.00 mL pH 6.5 phosphate buffer, the DPV curves were recorded from −0.3 to 0.6 V after 3-min accumulation. The results are listed in Table 4 and the recovery is satisfactory, indicating that the proposed method for simultaneous determination of AA, DA and UA is accurate and has promising application.

## Conclusions

A simple strategy to prepare carbon sensing materials with different electrochemical reactivity has been developed. The particle size of hard template has big impacts on the porous structure and BET surface area of the resulting carbon materials, and consequently affects the electrochemical properties of porous carbon such as response area, electron transfer ability and adsorption capacity. The constructed sensing interfaces using porous carbon exhibit strong enhancement effects toward the oxidation of AA, DA and UA, and greatly increase their oxidation signal. The largely-separated oxidation wave and the greatly-increased oxidation signals manifest that the prepared porous carbon is qualified for constructing a highly-sensitive detection platform for AA, DA and UA.

## Methods

### Materials

All chemicals were of analytical grade and used as received. AA, DA, UA were obtained from Aladdin Industrial Corporation (Shanghai, China). The standard solution of 1.00 M AA, 0.01 M DA and 0.01 M UA were individually prepared using ultrapure water. Starch and DMF were purchased from Sinopharm Chemical Reagent Company (Shanghai, China). ZnO nanoparticles with diameters of 15, 30, 50 and 100 nm were obtained from Nanjing Emperor Nano Material Co. Ltd. (Nanjing, China). Ultrapure water (18.2 MΩ) was obtained from a Milli-Q water purification system and used throughout.

### Instrumentation

Electrochemical experiments were carried out on a CHI 660D electrochemical workstation (Chenhua Instrument, Shanghai, China). A conventional three-electrode system, consisting of a glassy carbon working electrode, a saturated calomel reference electrode (SCE) and a platinum wire auxiliary electrode, was employed. Scanning electron microscopy (SEM) characterization was conducted with a Quanta 200 microscope (FEI Company, Netherlands). Transmission electron microscopy (TEM) image was measured using a Tecnai G220 microscope (FEI Company, Netherlands). X-ray diffraction (XRD) patterns were measured using a X’Pert PRO diffractometer, operating with Cu kα_1_ radiation in the 2θ scan range from 10° to 80° (Panalytical Company, Netherlands). Raman spectra were acquired on a LabRAM HR800 confocal Raman microscopy using 532 nm laser (Horiba Jobin Yvon, France). Nitrogen adsorption-desorption isotherms were obtained using a NOVA 1200 instrument (Quantachrome Corporation, USA).

### Preparation of porous carbon materials

Porous carbon samples were prepared using starch as the precursor and different-sized ZnO nanoparticles as the hard template. Firstly, 5.0 g of starch was dissolved into the mixture of 20.0 mL of alcohol and 40.0 mL of ultrapure water. After that, 5.0 g ZnO particles with diameter of 15, 30, 50 and 100 nm were individually added, and the mixture was dried at 80 °C under vigorous agitation. The resulting composites were heated to 800 °C at speed of 10 °C min^−1^, and then carbonized at 800 °C for 2 h under flowing nitrogen (99.99%). After that, the ZnO template was removed using 0.1 M HCl, and washed using ultrapure water for three times. After being dried at 80 °C in oven, the porous carbon samples were obtained, and individually denoted as PC-15, PC-30, PC-50 and PC-100.

### Construction of sensing interfaces

5.0 mg of the prepared porous carbon materials were exactly weighed, and added into 5.0 mL of DMF. After 1-h ultrasonication, stable suspensions with concentration of 1.0 mg mL^−1^ were achieved. Prior to modification, the glassy carbon electrode (GCE) with diameter of 3 mm was polished with 0.05 μm alumina slurry, and ultrasonically washed using ultrapure water to give a clean surface. Finally, 5.0 μL of suspension was added onto the surface of GCE, and dried under an infrared lamp in air. The constructed sensing interface was described as PC-15/GCE, PC-30/GCE, PC-50/GCE and PC-100/GCE.

## Additional Information

**How to cite this article**: Cheng, Q. *et al.* Morphology-dependent Electrochemical Enhancements of Porous Carbon as Sensitive Determination Platform for Ascorbic Acid, Dopamine and Uric Acid. *Sci. Rep.* **6**, 22309; doi: 10.1038/srep22309 (2016).

## Supplementary Material

Supplementary Information

## Figures and Tables

**Table 1:** Values of *Q*_*ads*_ on different sensing interfaces (μC).

Species	GCE	PC-15/GCE	PC-30/GCE	PC-50/GCE	PC-100/GCE
AA	0.846	1.947	3.113	2.307	1.35
DA	0.348	0.711	2.789	1.342	0.595
UA	0.512	3.049	6.371	5.348	2.535

**Table 2:** Analytical properties for AA, DA and UA using PC-30/GCE.

Analytes	Linear range	Regression equation	Detection limit
AA	10.0–3000.0 μM	*I*_pa_ (μA) = 0.0282 *C* (R = 0.999)	3.6 μM
DA	0.05–25.0 μM	*I*_pa_ (μA) = 2.867 *C* (R = 0.997)	15 nM
UA	0.05–30.0 μM	*I*_pa_ (μA) = 6.016 *C* (R = 0.999)	10 nM

**Table 3:** Performance comparisons of electrochemical sensing platforms for AA, DA and UA.

Sensing platform	Linear range (μM)			Sensitivity (μA μM^−1^)			Ref.
	AA	DA	UA	AA	DA	UA	
CuNPs/p-TAOX^a^/GCE^g^	240.0–750.0	0.60–21.6	4.00–103.0	0.030	1.300	0.203	21
Fe_3_O_4_@Au-S-Fc/GS-chitosan^b^/GCE^h^	6.0–350.0	0.50–50.0	1.00–90.0	0.030	0.290	0.150	22
GO-PAN^c^/GCE^i^	150.0–1050.0	1.00–40.0	3.00–26.0	0.008	0.265	0.209	23
N-S-PC^d^/GCE^i^	50.0–2000.0	0.10–50.0	0.10–50.0	0.016	1.352	1.355	24
CTAB/rGO/ZnS^e^/GCE^i^	50.0–1000.0	1.00–500.0	1.00–500.0	0.011	0.146	0.094	25
PtNSs/C_60_^f^/GCE^i^	10.0–1800.0	0.50–2.50	9.50–1187.0	0.008	0.164	0.014	26
PC-30/GCE^i^	10.0–3000.0	0.05–25.0	0.05–30.0	0.028	2.867	6.016	This work

^a^Cu-nanoparticles incorporated overoxidized-poly(3-amino-5-mercapto-1,2,4-triazole).

^b^phenylethynyl ferrocene thiolate modified Fe_3_O_4_@AuNPs coupling with graphene sheet/chitosan.

^c^Graphene oxide-templated polyaniline.

^d^Nitrogen and sulfur dual-doped porous carbon.

^e^Hexadecyl trimethyl ammonium bromide (CTAB) functionalized reduced graphene oxide/zinc sulfide nanocomposite.

^f^Platinum nanosheets/fullerene.

^g^GCE with diameter of 4 mm.

^h^The diameter is unknown.

^i^GCE with diameter of 3 mm.

**Table 4:** Detection of UA, DA and AA in blood serum samples.

Samples	Species	Detected (μM)	Added (μM)	Found (μM)	RSD (%)	Recovery (%)
Serum 1	AA	0	250.0	244.3	3.9	97.7
	DA	0	1.50	1.61	2.6	107.3
	UA	27.16	10.00	36.45	4.3	92.9
Serum 2	AA	0	500.0	485.1	1.9	97.1
	DA	0	3.00	2.86	6.7	95.3
	UA	17.77	15.00	32.89	4.8	100.8
