# Supplementary material for: Morphology-dependent Electrochemical Enhancements of Porous Carbon as Sensitive Determination Platform for Ascorbic Acid, Dopamine and Uric Acid
Source: Sci Rep. 2016 Feb 29;6:22309. doi: 10.1038/srep22309 (PMC4770411; doi:10.1038/srep22309)
Supplement: Supplementary Information [file srep22309-s1.pdf]

Supplementary materials

Morphology-dependent Electrochemical Enhancements of Porous Carbon as Sensitive Determination Platform for Ascorbic Acid , Dopamine and Uric Acid

Qin Cheng [a], Liudi Ji [a] , Kangbing Wu [a*], Weikang Zhang [b*]

[a] Key Laboratory for Material Chemistry of Energy Conversion and Storage, Ministry of Education, School of Chemistry and Chemical Engineering, Huazhong University of Science and Technology, Wuhan 430074, China

[b] Department of Gastrointestinal Surgery, Union Hospital, Tongji Medical College, Huazhong University of Science and Technology, Wuhan, 430022, China

* Corresponding author. K.W. Wu (kbwu@hust.edu.cn), W.K. Zhang (China.Weikangzhang11@163.com)

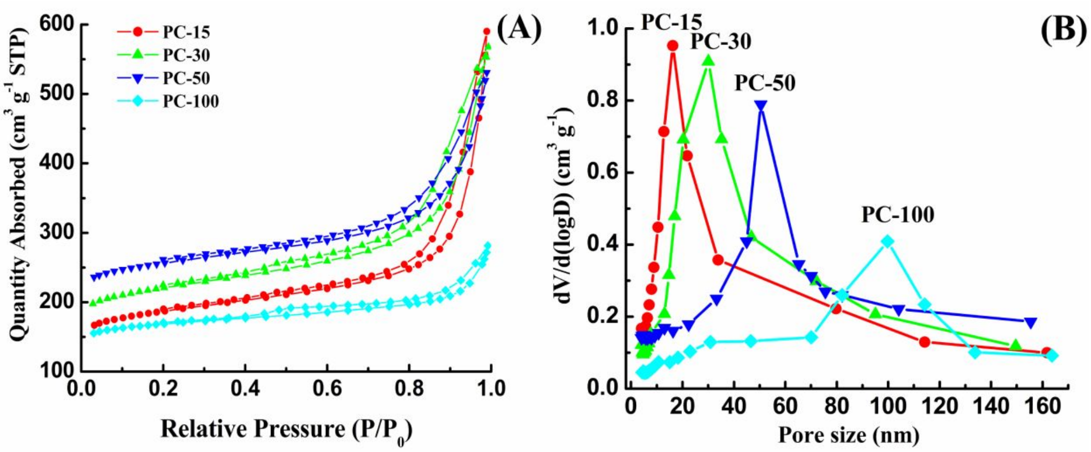

Figure S1. Nitrogen physisorption isotherms (A) and pore size distributions (B) of porous carbon samples.

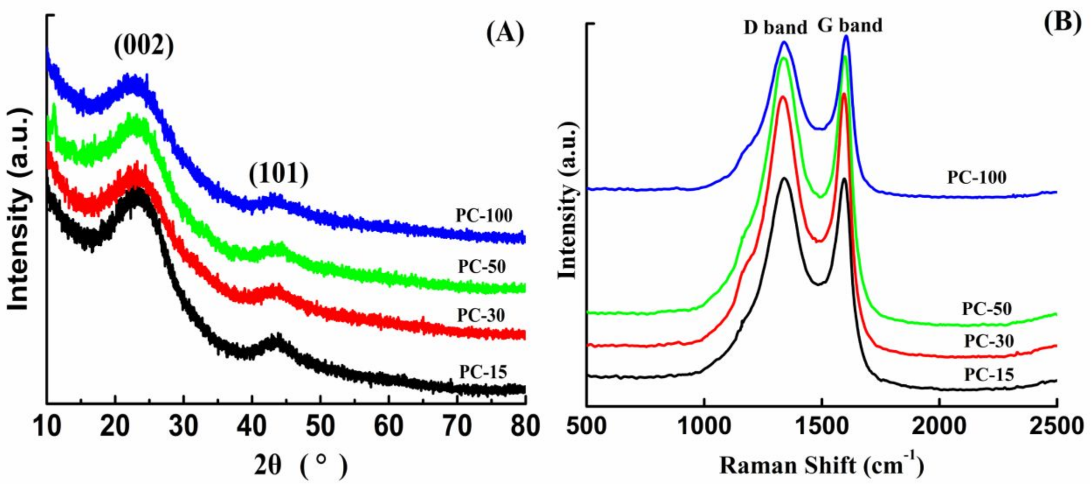

Figure S2. XRD patterns (A) and Raman spectra (B) of porous carbon materials.
